# Insights into photoacoustic speckle and applications in tumor characterization

**DOI:** 10.1016/j.pacs.2019.02.002

**Published:** 2019-04-05

**Authors:** Eno Hysi, Muhannad N. Fadhel, Michael J. Moore, Jason Zalev, Eric M. Strohm, Michael C. Kolios

**Affiliations:** a Department of Physics, Ryerson University, Toronto, ON, Canada; b Institute for Biomedical Engineering, Science and Technology, Li Ka Shing Knowledge Institute, Keenan Research Centre, St. Michael’s Hospital, Toronto, ON, Canada; c Department of Mechanical and Industrial Engineering, University of Toronto, Toronto, ON, Canada; d Translational Biology and Engineering Program, Ted Rogers Centre for Heart Research, Toronto, ON, Canada

**Keywords:** Photoacoustic speckle, Ultrasound speckle, Spatial resolution, Autocovarience function, Tumor vasculature, In-vivo imaging, Vascular trees

## Abstract

In ultrasound imaging, fully-developed speckle arises from the spatiotemporal superposition of pressure waves backscattered by randomly distributed scatterers. Speckle appearance is affected by the imaging system characteristics (lateral and axial resolution) and the random-like nature of the underlying tissue structure. In this work, we examine speckle formation in acoustic-resolution photoacoustic (PA) imaging using simulations and experiments. Numerical and physical phantoms were constructed to demonstrate that PA speckle carries information related to unresolved absorber structure in a manner similar to ultrasound speckle and unresolved scattering structures. A fractal-based model of the tumor vasculature was used to study PA speckle from unresolved cylindrical vessels. We show that speckle characteristics and the frequency content of PA signals can be used to monitor changes in average vessel size, linked to tumor growth. Experimental validation on murine tumors demonstrates that PA speckle can be utilized to characterize the unresolved vasculature in acoustic-resolution photoacoustic imaging.

## 1 Introduction

Photoacoustic (PA) imaging allows for high contrast visualization of the vasculature [1], pharmacokinetic drug distribution [2] and neuronal functional connectivity [3]. Clinical applications include melanoma detection [4], assessment of Crohn’s disease [5], breast imaging [6] and cancer treatment monitoring [[7], [8], [9], [10], [11]]. PA images are affected by the light illumination geometry, choice of optical wavelength and ultrasonic detection characteristics. Speckle is a ubiquitous property of all coherent modalities such as laser imaging [12], ultrasound (US) [13] and optical coherence tomography [14]. Due to the similarities between the two techniques, PA speckle is analogous to US speckle, which results from the spatiotemporal superposition of waves backscattered from randomly positioned objects within the imaging transducer’s resolution volume. At clinical frequencies, ultrasound speckle is considered fully developed for a scatter density of >100/mm^3^ or, more generally, when there are at least 10 scatterers per transducer resolution volume [15]. In US, acoustic impedance mismatches from sub-resolution inclusions scatter the incident US pulse. The scatterer’ spatial position gives rise to phase differences which produce fluctuations in image intensity (i.e. speckle) due to wave superposition. Such patterns are deterministic and described by first and second-order statistics. As long as the position of the scattering sources does not change with time, the speckle patterns remains unchanged. Therefore, speckle encodes spatial information about the scatterers. The distribution of signal amplitudes (first-order statistics) is independent of the transducer aperture [16]. Second-order statistics, e.g. the spatial autocovariance function (ACVF), provide an estimate of speckle size, which in turns depends on pulse bandwidth, beamwidth, transducer f-number, working distance and consequently, the spatial resolution of the US system [17].

The physics of PA acoustic wave propagation is the same as US wave propagation. The main difference between the signals produced by both modalities lies in the broadband nature of PA imaging. Theoretically, PA signals are infinitely broadband [18] while the frequency content of backscattered US signals is only a subset of the frequencies emitted in the initial transmit pulse from the transducer [19]. While PA signals are also bandpassed by the receiving transducer, they contain a larger range of frequencies than in the case of US signals. This might be desirable in approaches involving frequency analysis of PA signals and images such as F-Mode [20] or radiofrequency spectroscopy [21]. For this reason, frequency-dependent attenuation, diffraction and the transducer focus, geometry, frequency and bandwidth affect PA images [22]. PA pressure waves from multiple absorbers also generate constructive and destructive interference [23]. At boundaries between media, constructive interference typically produces a high amplitude signal, while both constructive and destructive interference occurs between boundaries. The signal from the boundary usually saturates the dynamic range of the resultant PA images giving the impression that acoustic resolution PA imaging is a speckle-free modality [[24], [25], [26]] even though speckle is present in applications involving non-resolvable absorbers [27,28]. The prominence of this boundary “edge-effect” is strongly influenced by the transducer properties, system response and absorber concentration. Our group has demonstrated through simulations [23,29] and experiments [[30], [31], [32]] that the signals giving rise to speckle are dependent on the reconstruction approach, spatial distribution of PA sources and the bandwidth of the ultrasound detector.

In US imaging, the “grainy” appearance of speckle makes it difficult to differentiate between different types of soft tissues which suffer from low contrast due to the narrow range in their mechanical properties. To overcome this limitation, quantitative ultrasound (QUS) has emerged as a system-independent approach relying on the analysis of the radiofrequency (RF) signals from the backscattered US waves [33]. QUS has been used to quantify scatterer shape, size, concentration, spatial organization or mechanical properties [34] and characterize blood [35] or breast cancer treatment response [36]. Given the parallels between US and PA imaging, it is hypothesized that the similar analysis techniques can be applied to extract quantitative parameters in PA without having to resolve the individual absorbing structures.

PA speckle has been largely considered to be a detrimental aspect of imaging and most illumination and detection geometries as well as image post-processing tools attempt to suppress its formation [1]. However, in cases where high resolution PA images cannot be attained, and speckle is present, the deterministic nature of speckle permits quantitative analysis of the underlying absorbers. In this work, we demonstrate the formation of PA speckle in limited-view transducer geometries and demonstrate how speckle patterns are affected by the imaging transducer properties. Theoretical and experimental evidence shows that PA speckle encodes structural information about non-resolvable absorbers. Lastly, we illustrate how tumor vascular development can be monitored in cases where PA speckle is present in acoustic-resolution PA imaging. In this paper, we establish the mathematical formulation of PA speckle in limited-view transducer geometries and demonstrate experimentally how speckle patterns are affected by imaging transducer properties. We also discuss how the structural information encoded in PA signals can be extracted by RF analysis of signals in the temporal and frequency domain and how this approach can be applied to monitor tumor vascular development.

## 2 Mathematical formulation of PA speckle

**Fig. 1:**
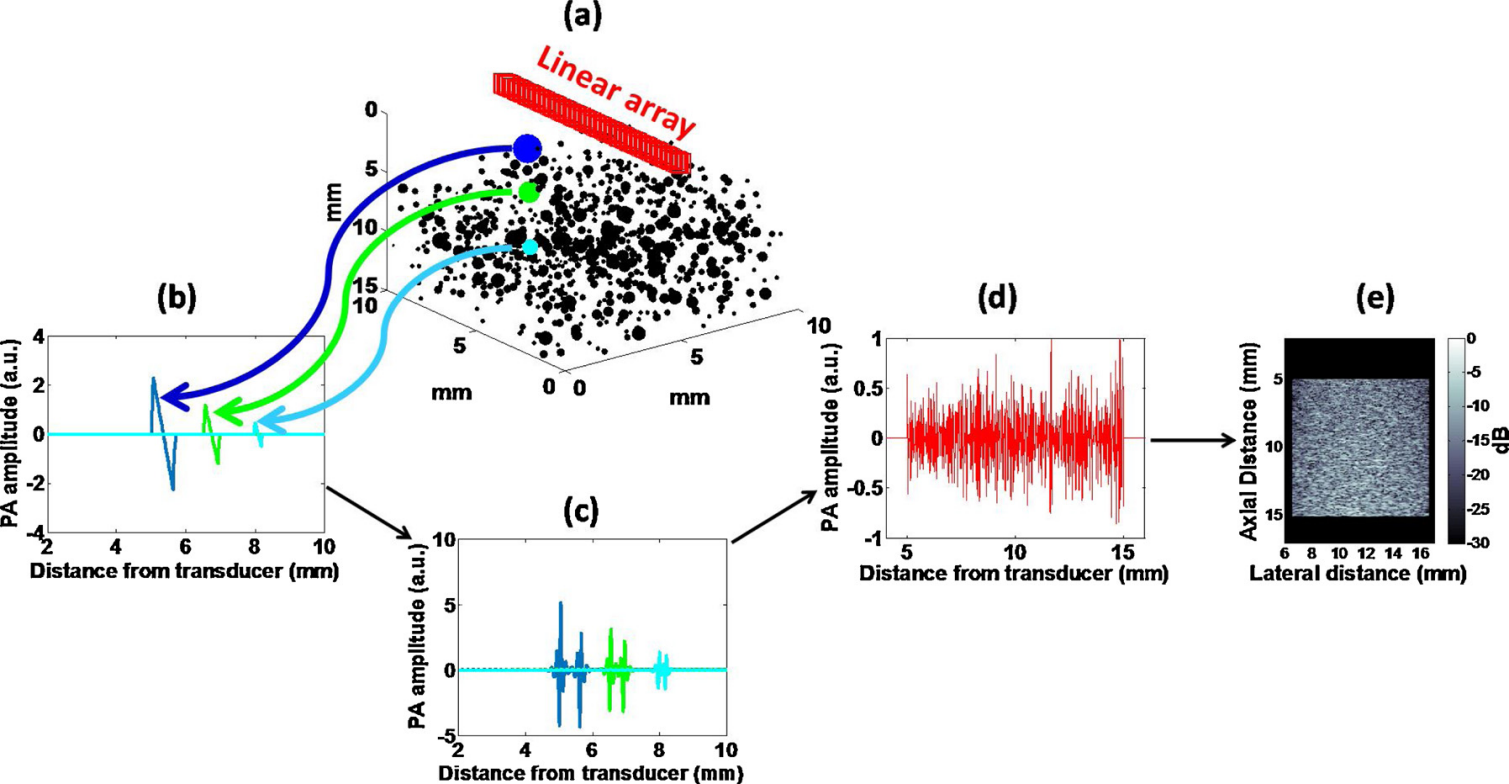
Formation of PA speckle. (a) 3D visualization of the geometry of a numerical phantom containing randomly positioned spherical absorbers of different diameter. (b) Typical non-bandlimited PA signals from absorbers of three different diameters. (c) Bandlimited version of PA signals shown in (b). The range of frequencies is determined by the ultrasound detector used. (d) Representative, beamformed PA RF line with contributions from all absorbers. (e) Resultant PA B-mode image obtained by delay-and-sum beamforming.

PA speckle arises from the linear superposition of the pressure waves generated as a result of the short-pulse, optical excitation of a medium consisting of optical absorbers. Fig. 1 shows a visual representation of PA speckle formation from a collection of randomly positioned absorbers (Fig. 1a) imaged with a linear array transducer with N elements. The mathematical formation of PA speckle begins with derivations of the time-dependent PA emission obtained by solving the wave equation for pressure p [18,37,38]. Using Green’s functions for spherical and cylindrical absorbers, the solution for the wave equation can be represented in terms of a velocity potential ϕ:

(1) $$\phi \left(r,t\right)=-\frac{\beta }{4\pi \rho C_{P}}\int_{0}^{t}dt^{\prime }\int g\left(r,t|r^{\prime },t^{\prime }\right)H\left(r^{\prime },t^{\prime }\right)dr^{\prime }$$

where, β is the thermal expansion coefficient, ρ is the density, $C_{P}$ is the heat capacity per unit mass, r is the spatial position, H is the heating function defined as the energy per unit volume and time t deposited by the incident radiation beam in the sample. The pressure is the first time derivative of the velocity potential, namely p=−ρ∂ϕ∂t (Fig. 1b). In our model, the time domain impulse response of the transducer as a function of center frequency is given by [29]:

(2) $$g_{T}\left(\varpi_{0},\xi ,t\right)=\frac{\xi }{\sqrt{2\pi }}\exp\left(-\frac{\xi^{2}t^{2}}{2}\right)\cos\left(\varpi_{0}t\right)$$

where, $\varpi_{0}$ is the center frequency, ξ is the −6 dB bandwidth. To account for the limited-view geometry, a directivity function $D\left(\alpha \right)$ was computed for each element as a function of angle α relative to the center of each transducer element [39]:

(3) $$D\left(\alpha \right)=\left|\frac{2J_{1}\left(kR\sin\alpha \right)}{kR\sin\alpha }\right|$$

where, $J_{1}$ is the first order Bessel function, k is the wavenumber and R is the transducer aperture. The bandlimited (BL) PA signal from M absorbers (Fig. 1c) is the temporal convolution of the directivity-corrected pressure from each absorber $p_{m}\left(r,t\right)$ with Eq. (2):

(4) $$p_{BL}\left(t\right)=\sum_{m}^{M}\left[p_{m}\left(r,t\right)\times D\left(\theta \right)\right]*g_{T}\left(\varpi_{0},\xi ,t\right)$$

In acoustic-resolution PA imaging using linear array transducers, the image is generated from the recorded PA signals using delay-and-sum beamforming by dynamically focusing to a point in the imaging space using $N_{A}$ sub-aperture elements. The $p_{n}^{BL}\left(t\right)$ generated from an absorbing source located at location $r_{m}$ will arrive at the $n^{th}$ transducer element at time $t=\left(\left|r_{n}-r_{m}\right|\right)/v_{s}$. At the ${\left(n+1\right)}^{th}$ element, the RF signal from the same source will be delayed by $\tau_{n}=\left(\left|r_{n+1}-r_{m}\right|-\left|r_{n}-r_{m}\right|\right)/v_{s}$ due to the path difference created by the spatial separation of adjacent elements. Signals received from the spatial position $r_{m}$ were compensated for the relative delays on sub-aperture $N_{A}$ and adding their contributions. The beamformed (BF), bandlimited PA RF line (Fig. 1d) then becomes:

(5) $$p_{BL}^{BF}\left(t\right)=\sum_{n=0}^{N_{A}-1}p_{n}^{BL}\left(t-\tau_{n}\right)$$

The summation/interference of all time-delayed PA signals is the source of PA speckle, as is the case in US imaging [13]. The phase of each wavelet is determined by the time delay, and thus spatial position, of each absorber within the transducer’s resolution volume. The summation of the wavelets contributes to an interference pattern. The interference pattern depends on the spatial distribution and physical properties of the absorbers and can be analyzed using speckle envelope statistics [40] and frequency-based techniques [36], as described in the next section. Following beamforming, the PA images are displayed using the logarithmically compressed Hilbert transform of the RF matrix at each scanning location. The final, reconstructed image shown in Fig. 1e is derived by taking the envelope of the RF signals using the Hilbert transform H:

(6) $$I\left(x,z\right)=20\log_{10}\left(A\right)=20\log_{10}\left|H\left\{p_{BL}^{BF}\left(x,z\right)\right\}\right|$$

The spatial coordinates $\left(x,z\right)$ are obtained using the transducer pitch and speed of sound in the lateral $\left(x\right)$ and axial $\left(z\right)$ directions, respectively.

## 3 Materials and methods

In this section, three analysis techniques will be introduced for probing the morphological properties of non-resolvable absorbing structures giving rise to speckle. Simulations of media containing spherical and cylindrical absorbers will be used to demonstrate the formation of PA speckle. Experimental phantoms containing non-resolvable absorbers will illustrate the effect of the imaging transducer spatial resolution on speckle size. As the primary source of the photoacoustic signals in tissues is red blood cells, simulations of vascular trees and PA images of mouse tumors will be used to illustrate changes in speckle characteristics in growing tumors.

### 3.1 Analysis methods

**Fig. 2:**
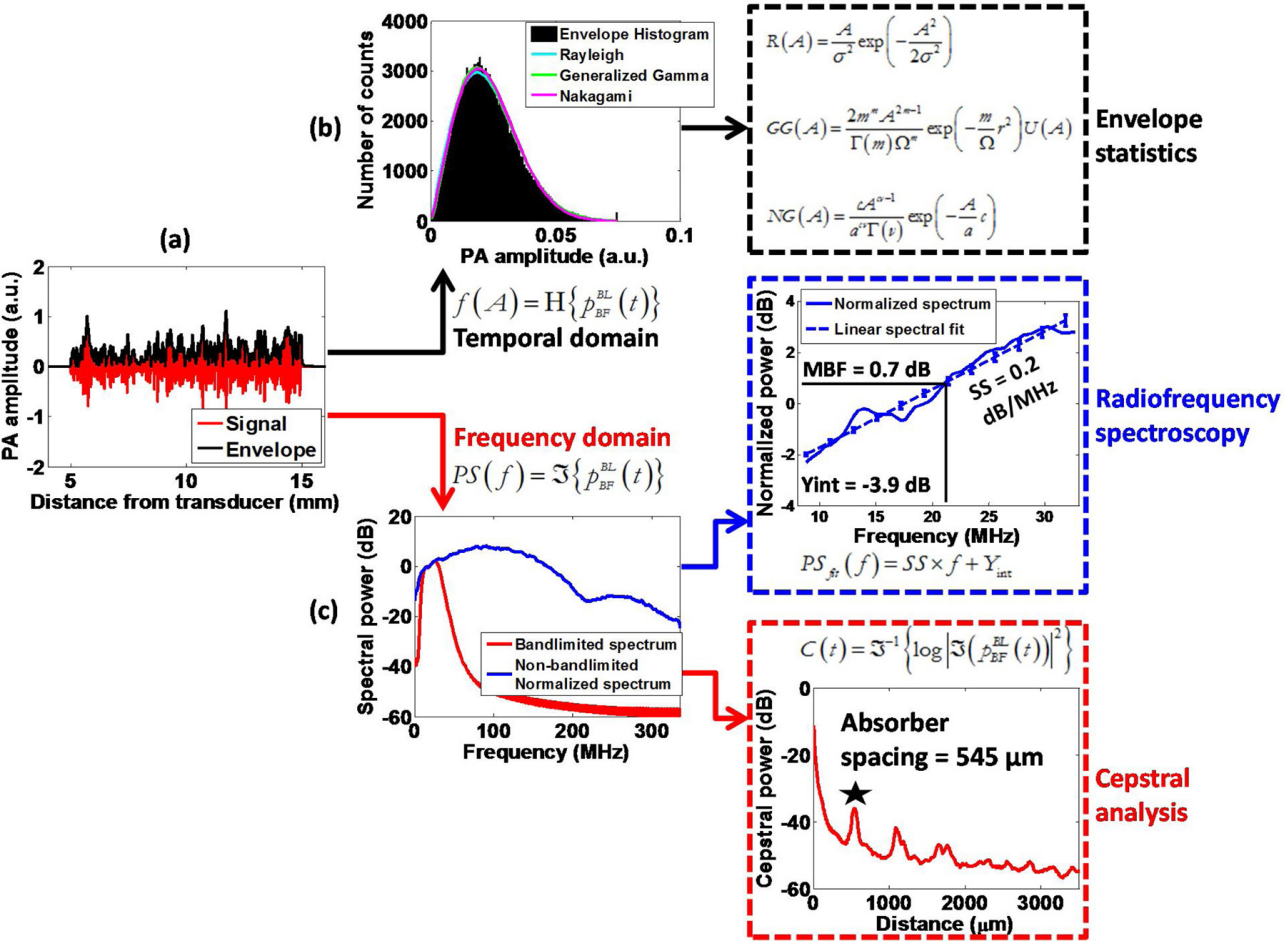
Overview of analysis techniques. (a) Representative PA RF signal (red) and its envelope (black). (b) Temporal domain analysis obtained through envelope statistics (top box). (c) Frequency domain analysis obtained through the Fourier transform of PA signals yields radiofrequency spectroscopy (middle box) and cepstral analysis (bottom box).

Fig. 2 shows an overview of the three techniques used in this paper for analyzing PA signals. Each method was applied to both simulations and experimentally acquired PA images containing speckle from spherical and cylindrical absorbers. The major distinction between the techniques was whether the signal analysis was performed in the temporal or frequency domain. In combination, these techniques will be used to evaluate the changes in PA images due to variations in the concentration and size of unresolvable absorbers (Sections 4.1.1 and 4.1.2) and identify periodic patterns present in PA images containing speckle (Section 4.1.3). Each analysis technique is described below:

(i) The envelope statistics method models the amplitude of the envelope of the RF signals by a statistical distribution. The distribution characteristics depend on the physical size, concentration, and spatial distribution of absorbing structures [40]. The amplitude of the envelope of the RF signals, A, for each RF signal in a phantom was compared to three statistical distributions: Rayleigh, Generalized Gamma, and Nakagami (Fig. 2b). Fully developed speckle was established when the ratio of the mean to the variance of the amplitude (also known as the speckle signal to noise ratio, SNR) is equal to 1.91 [41]. The fit parameters of each distribution were recorded as a function of absorber size and concentration.
(ii) The radiofrequency spectroscopy technique is based on the analysis of the frequency content of the PA signals which changes based on the size and shape of the absorbing structures [18]. The spectral slope (SS) of the normalized or non-bandlimited power spectrum (Fig. 2c, middle box) was computed by linear regression within the -6 dB bandwidth of the transducer for a range of absorber sizes and increasing absorber size polydispersity. The normalized PA power spectra were obtained either from the non-bandlimited PA frequency spectra in the case of the simulations, or by normalizing the power spectra by spectra acquired from a reference signal for experiments. This approach is similar to what is used in ultrasound tissue characterization [42]. A straight line was fit to the resultant normalized spectra within the -6 dB bandwidth of the transducer in order to extract the spectral parameters:

(7) $$PS_{fit}(f)=SS\times f+Y_{\mathrm{int}}$$

where, $PS_{fit}$ is the result of performing linear regression on the normalized spectra, SS is the spectral slope measured in dB/MHz and $Y_{\mathrm{int}}$ is the y-intercept of the fit measured in dB. The average SS was computed for all the spectra included in a ROI, for various of absorber sizes and increasing absorber size polydispersity.

• The cepstral analysis technique uses the frequency content of the RF signals to estimate the absorber spacing [43]. This technique determines the temporal location of the dominant (first) cepstral peak which can be converted to a physical distance by accounting for the speed of sound within the medium, 1540 m/s in this case (Fig. 2c, bottom box). The Euclidean norm was used to calculate the true absorber spacing from spatial coordinates.

For each technique, the PA B-mode images obtained from Eq. (6) were divided into overlapping (75%), square-shaped ROIs whose size was ˜10 ultrasonic wavelengths (estimated from the center frequency of the imaging transducer). The average size of the speckle in a PA B-mode image was calculated by the 2-D ACVF which is a descriptor of the spatial correlation of texture [17]. In the frequency domain, the ACVF is given by:

(8) $$ACVF\left(\varpi_{x},\varpi_{z}\right)=U\left(\varpi_{x},\varpi_{z}\right)U^{*}\left(\varpi_{x},\varpi_{z}\right)={\left|U\left(\varpi_{x},\varpi_{z}\right)\right|}^{2}$$

where, $U\left(\varpi_{x},\varpi_{z}\right)$ is the 2-D Discrete Fourier transform of the PA image obtained from Eq. (7) which contained within the ROI over the spatial frequencies $\varpi_{x}$ and $\varpi_{z}$, and $U^{*}\left(\varpi_{x},\varpi_{z}\right)$ is its complex conjugate. After transforming the $ACVF\left(\varpi_{x},\varpi_{z}\right)$ back to the spatial domain through an inverse Fourier transform, the cross-sections of the corresponding 2-D ACVF in the axial and lateral directions is taken to calculate the respective speckle sizes.

### 3.2 Simulations

#### 3.2.1 Modeling spherical absorbers

Numerical phantoms of dimensions 10 × 10 × 10 mm were constructed within a 1000 mm^3^ region of interest (ROI). Spherical beads of radius a were randomly positioned within the phantom. PA signals were computed from the time derivative of Eq. (1). The transducer modeled was the VevoLAZR (Fujifilm-VisualSonics Inc., Toronto, Canada) which is a 21 MHz linear array probe (LZ250 model, 256 elements, 90 μm pitch, 9–33 MHz −6 dB bandwidth, 5.44 × 10^6^ μm^3^ resolution volume) [9,44,45]. It was positioned 5 mm above the phantom. A total of 86 distinct, numerical phantoms were simulated to test analysis techniques described in Section 3.3:

(i) The envelope statistics phantoms (27 phantoms) were simulated by changing the size (5–60 μm) and the concentration of beads (0.1, 1 and 10 beads per resolution volume).
(ii) The radiofrequency spectroscopy phantoms (50 phantoms) were simulated by changing the bead radius from 5 to 135 μm to test the effect of size and polydispersity (achieved by changing the standard deviation (θ parameter) of a Generalized Gamma distribution for bead size).
(iii) The cepstrum analysis phantoms (11 phantoms) were simulated with different populations of periodic and random absorbing structure (defined as the grid-to-random or G:R ratio). The beads were either placed in a 3D grid of defined spacing (100:0% ratio) or partially randomized (50:50% and 15:85% ratios). For the latter, the beads concentration ranged from 8 to 1469/mm^3^.

#### 3.2.2 Modeling cylindrical absorbers

A 3D computational vascular network model was generated to investigate the impact of the geometrical arrangement of cylindrical absorbers (blood vessels) on the formation of PA speckle. The approach is based on a fractal geometrical model described elsewhere [38,46,47]. Briefly, the network begins with a parent vessel bifurcating to give rise to two daughter branches which bifurcate further. Using cylindrical segments, the vascular trees evolved successively down to the arteriole level (diameter ˜20 μm). For the purposes of this study, chaotic tumor vasculature [48] was modeled by randomly changing the branching angle between 25–140° [47]. It was not our intention to model the complex vascular morphology of a tumor but rather to investigate the effects that increasing the vessel diameter, branching order and vessel length might have on PA speckle signals. These changes in vessel characteristics are well documented in animal models of tumor growth [49,50].

The simulation parameters were based on histological measurements of vessel size from in-vivo mouse tumors at 7 and 14 days post-inoculation (see Section 3.3.2) [8], [9]. The following simulation parameters were used to achieve a mean diameter of all cylinders as close to the experimentally measured vessel size:

(i) Day 7 tumors: 184 μm parent vessel diameter; 0.75 mm parent vessel length; 37.8 μm mean diameter; 360 μm mean vessel length; branching order of 9; 508 total vessels.
(ii) Day 14 tumors: 460 μm parent vessel diameter; 1 mm parent vessel length; 48.2 μm mean diameter; 352 μm mean vessel length; branching order of 12; 4092 total vessels.

For every cylinder in the vascular tree, the PA signals were computed by solving Eq. (1) for the radii r and lengths L listed above. For these simulations, the transducer modeled was a VevoLAZR 40 MHz linear array probe (LZ550 model, 256 elements, 50 μm pitch, 9–50 MHz -6 dB bandwidth).

### 3.3 Experiments

#### 3.3.1 Construction and imaging of physical phantoms

To study the dependence of PA speckle dimensions on the properties of the imaging transducer, three physical phantoms were constructed and imaged with four transducers (5–400 MHz) using three different PA imaging systems, the Ultrasonix RP linear array, the VevoLAZR US/PA linear array, and the SASAM PA microscope (Supplementary Fig. 1). The dimension of the phantoms, the acoustic properties of the imaging transducers and the phantom preparation methods are summarized in Supplementary Fig. 2. The optical absorbers for each system were either black glass (5 MHz transducer) or black polystyrene (40/200/400 MHz transducer) beads. The phantoms were constructed so that there were at least 10 beads per resolution volume of each transducer. For images acquired using the 5, 40, and 200/400 MHz transducers, the speckle size was estimated by computing a single ACVF for 305, 315, and 120 different overlapping ROIs within the image, respectively. All ACVFs from the same image were then averaged to obtain the mean speckle size. For each phantom, envelope statistics techniques were applied to obtain the SNR and the Nakagami m parameter. For the 40 MHz transducer, each of the analysis techniques described in Section 3.1 were performed for two different spherical absorber diameters (3.5 and 15 μm).

#### 3.3.2 In-vivo imaging of tumors

All experimental protocols were approved by the University Health Network (Toronto, Canada) Animal Care Committee. A murine breast cancer cell line (EMT-6) was inoculated subcutaneously on the footpad of BALB/c mice. Volumetric PA images of the tumors were acquired using 750 nm illumination with the VevoLAZR system (Fujifilm-VisualSonics, Toronto, Canada). Imaging was performed 7 days (n = 3 mice) and 14 days (n = 4 mice) post-inoculation with mice sacrificed after imaging. CD31 histological staining was used to compute the average vessel size at each imaging time point for comparisons with the vascular trees (see Section 3.1.2). A PA reference phantom consisting of black carbon beads (1–12 μm diameter) was used as a measure of the system dependencies and to remove the system response for RF spectroscopy [9], as described in Section 3.1. The size of speckle (estimated from the 2D ACVF) from images of in-vivo tumors as well as simulated vascular trees were compared to the transducer spatial resolution.

## 4 Results and discussion

### 4.1 Probing the structural properties of non-resolvable, spherical absorbers

#### 4.1.1 Envelope statistics

**Fig. 3:**
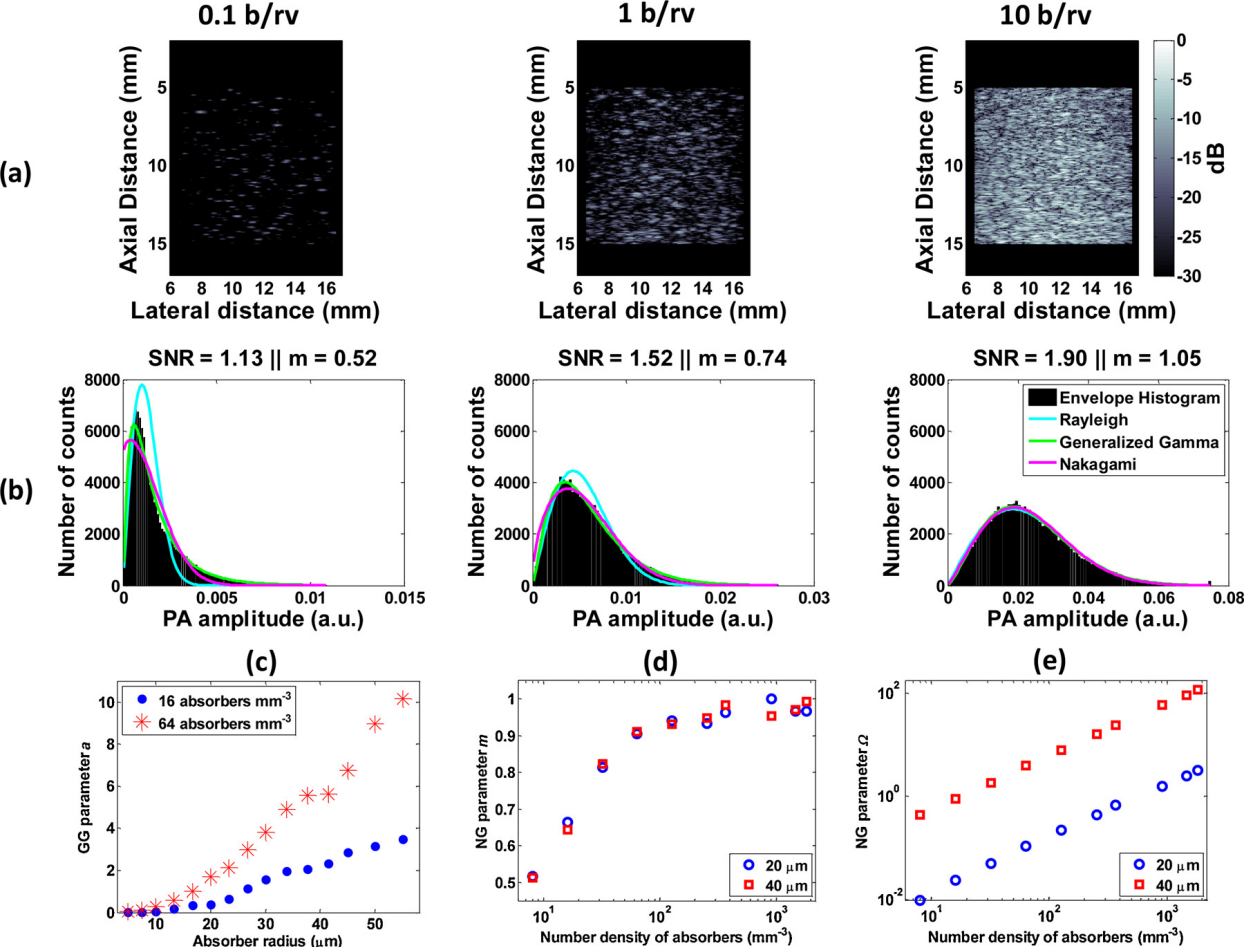
Simulation results for the envelope statistics method. (a) PA Bmode images of 5 μm spherical bead phantoms for increasing b/rv. (b) Corresponding PA signal envelope histograms fitted to the Rayleigh, Generalized Gamma (GG) and Nakagami (NG) statistical distributions. (c) GGa, (d) NG m and (e) NG Ω parameters plotted as a function of size and concentration.

Fig. 3a shows PA B-mode images from collections of randomly positioned, spherical absorbers for increasing bead concentration. The images are normalized to the maximum image pixel amplitude of the image containing approximately 10 beads per resolution volume (b/rv) of the imaging transducer (5.44 × 10^6^ μm^3^). As absorber concentration increased, the PA signal amplitude increased, resulting in a shift of the envelope histograms to the right (Fig. 1b). The envelope histograms can be used to assess the presence of fully developed speckle by relying on the either SNR metric and/or the fit to the Rayleigh probability density function (PDF) [40]. Fully developed speckle was observed as the speckle SNR approached the value of 1.91 with increasing bead concentration (Fig. 3b). The other two statistical models, the Generalized Gamma and Nakagami provide better fits than Rayleigh in cases of lower number densities (0.1 and 1 b/rv) and become identical to Rayleigh for ≥10 b/rv (SNR = 1.91). These results agree well with those obtained with envelope statistical analysis in US imaging [51] and support the assertion that speckle statistics can be used to characterize acoustic-resolution PA images. Studies from US imaging show that the Generalized Gamma a parameter is sensitive to scatterer size and concentration. Additionally, the Nakagami m parameter approaches 1 for fully developed speckle, independent of scatterer size [52]. Our simulations demonstrate that the same trend holds when considering PA emission from uniformly illuminated spheres. Specifically, the a parameter increases monotonically with absorber radius (Fig. 3c) and the m parameter becomes 1 for >100 absorbers/mm^3^ (Fig. 3d). The Ω parameter (Fig. 3e) increases 36x for the range of number densities examined. In US imaging, the Nakagami fit parameters have been used to characterize the scatter properties in malignant and benign breast tumors [52]. We hypothesize that application of similar statistical analysis techniques to experimentally acquired PA data can be used for characterizing changes during vascular-targeted cancer treatments [53].

#### 4.1.2 Radiofrequency spectroscopy

**Fig. 4:**
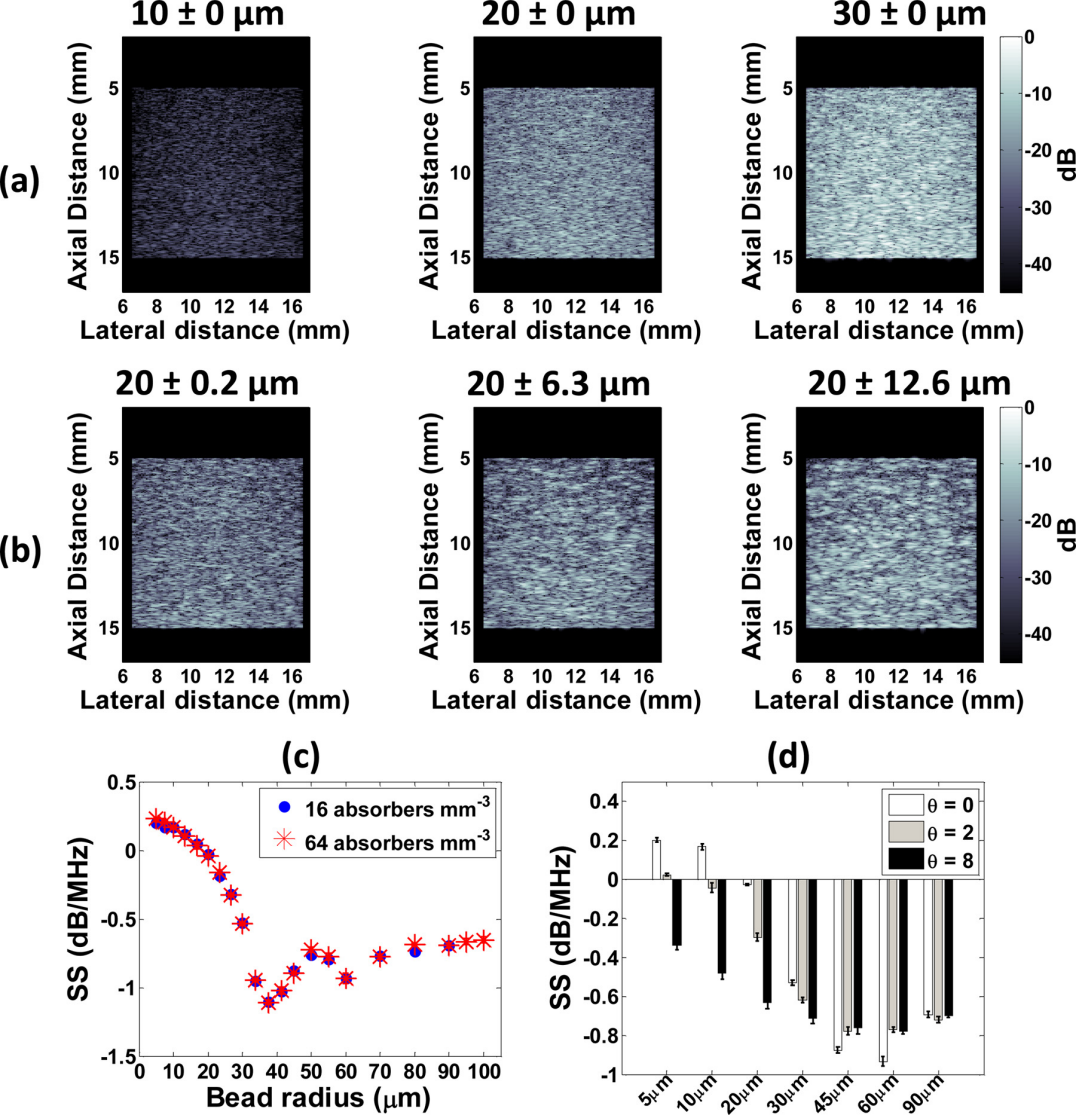
Simulation results for the radiofrequency spectroscopy method. PA B-mode images for (a) monodisperse and (b) polydisperse collection of beads at a concentration of 10 beads per resolution volume. SS as a function of (c) bead radius and concentrations and (d) polydispersity (controlled by the θ parameter).

Given that PA signal amplitude is affected by absorber morphology, size, concentration, optical absorption and laser fluence [22], it can be difficult to isolate the impact that any one of these factors has when imaging biological tissue [54]. Here, we investigate a subset of these parameters (size and concentration) in a well-controlled simulation. Fig. 4a and b show PA speckle in simulated PA B-mode images for various absorber sizes and degree of polydispersity. The mean image intensity increased with size (14.5× from 10 to 30 μm) and polydispersity (2× from 0.2–12.6 μm). Fig. 4c shows the PA spectral slope (SS) as a function of bead radius for two different absorber concentrations. Increasing absorber size decreases the SS regardless of concentration, suggesting that the PA RF spectra contain information about the absorber size. This is consistent with previous studies in which the SS was used to quantify microstructure in biological tissue and for red blood cell aggregation in blood suspensions [32,[55], [56], [57]].

These results suggest that the PA SS can be used to monitor changes in size without resolving absorbing structures. This is in agreement with previous PA studies at lower ultrasonic frequencies [58]. Fig. 4c reveals that beyond 30 μm, the SS remains constant with increasing size for the frequencies considered in these simulations. Thus, it may be possible to determine when the absorber radius is above or below a certain threshold (30 μm in this case) by computing the SS parameter from an experimentally acquired PA B-mode image. The SS curve flattening for larger sphere size seen in Fig. 4c is due to the relation of absorber size to the wavelength of the detected US wave. In US imaging, Rayleigh and Faran scattering regimes are defined for scatterering structures whose size is smaller and similar to/larger than the US wavelength, respectively [19]. Due to the broadband nature of PA signals, frequencies from equivalent regimes are present in PA imaging. For ka>1 (where k=2πf/c is the ultrasonic wavenumber of the detected ultrasound as determined by the transducer characteristics), the SS does not significantly change with absorber size and no variations in spectral features (minima and maxima) are apparent. This is seen in Fig. 4d where the PA SS becomes more negative for increasing polydispersity, but only up to 30 μm. In biological tissues, polydispersity exists at multiple biological length scales [21] but is difficult to identify in PA B-mode images alone (Fig. 4b). The PA SS provides a tool for assessing the presence of polydispersity (observing a decreasing SS as a function of size) and quantifying its degree (changing magnitude of slope) with potential applications in monitoring vascular therapies longitudinally [[7], [8], [9], [10], [11]].

#### 4.1.3 Cepstral analysis

**Fig. 5:**
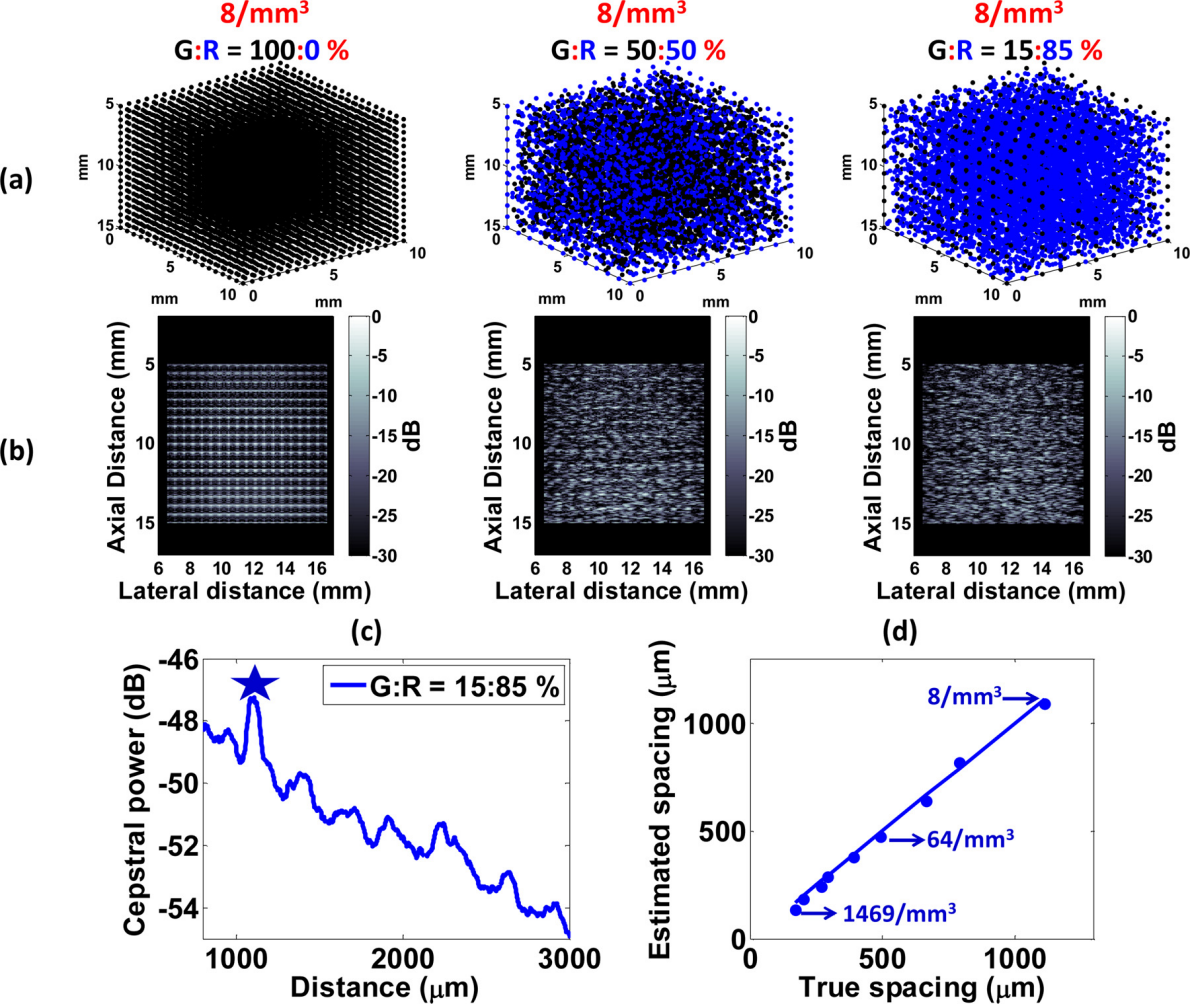
Simulation results using the cepstral analysis method. (a) Geometries of phantoms for various grid (G) to random (R) ratio and (b) their corresponding PA B-mode images. (c) Power cepstrum of the 15:85% phantom with star denoting the location of the first peak. (d) Comparison of the estimated absorber with the true spacing for various number densities.

Fig. 5a shows the geometries of numerical phantoms with various percentages of regularly spaced 10 μm absorbers (denoted by the grid-to-random or G:R ratio). The number density of absorbers was 8/mm^3^. B-mode images are shown in Fig. 5b. When all the absorbers are arranged in a grid pattern (i.e. G:R = 100:0%), their periodicity enhances the PA signal at specific spatial positions of the absorber (or, equivalently, absorbers separated by specific distances). It should be noted that the apparent size of the beads in Fig. 5b is not an accurate representation of their physical dimensions (10 μm) but determined by the transducer’s point spread function.

As the percentage of randomly positioned absorbers increases, the PA image begins to look like the B-modes shown in Fig. 3, Fig. 4 but fully developed speckle does not form (SNR < 1.91). A challenge with such images is identifying whether the underlying tissue structure contains periodicity in the spatial arrangement of absorbers. This is of particular interest in imaging the vascular development in tumors [53]. The tumor vasculature does not have the structural organization characteristic of normal tissue. PA imaging of whole tumors could in principle be used to quantify such changes in organization in cases where individual vessels cannot be fully resolved.

Fig. 5c shows the average power spectrum when 15% of all absorbers are arranged periodically. The location of the first cepstral peak (denoted by the star) is a measure of the most commonly occurring spacing between absorbers [43]. In the 15:85% case, the periodic absorbers were spaced approximately 1 mm apart, with randomly positioned absorbers occupying the space between. The spacing calculated through spectral analysis in Fig. 5c agrees well with the a priori known grid spacing. The cepstral-estimated absorber spacing was compared for various absorber concentrations ranging from 8/mm^3^ to 1469/mm^3^ (fully developed speckle). The results, shown in Fig. 5d, suggest that the technique can be used to estimate the most predominant spacing of an ensemble of sub-resolution absorbers (without having to resolve individual constituents). This demonstrates the potential of speckle cepstral analysis in biomedical applications such as monitoring therapeutic microwave lesions in the liver [59]. We believe that this approach can find applications in PA imaging using limited-view geometries for understanding the physical properties of various biological tissues such cancerous tumors [10] and liver [28].

### 4.2 Experimental evidence for PA speckle

**Fig. 6:**
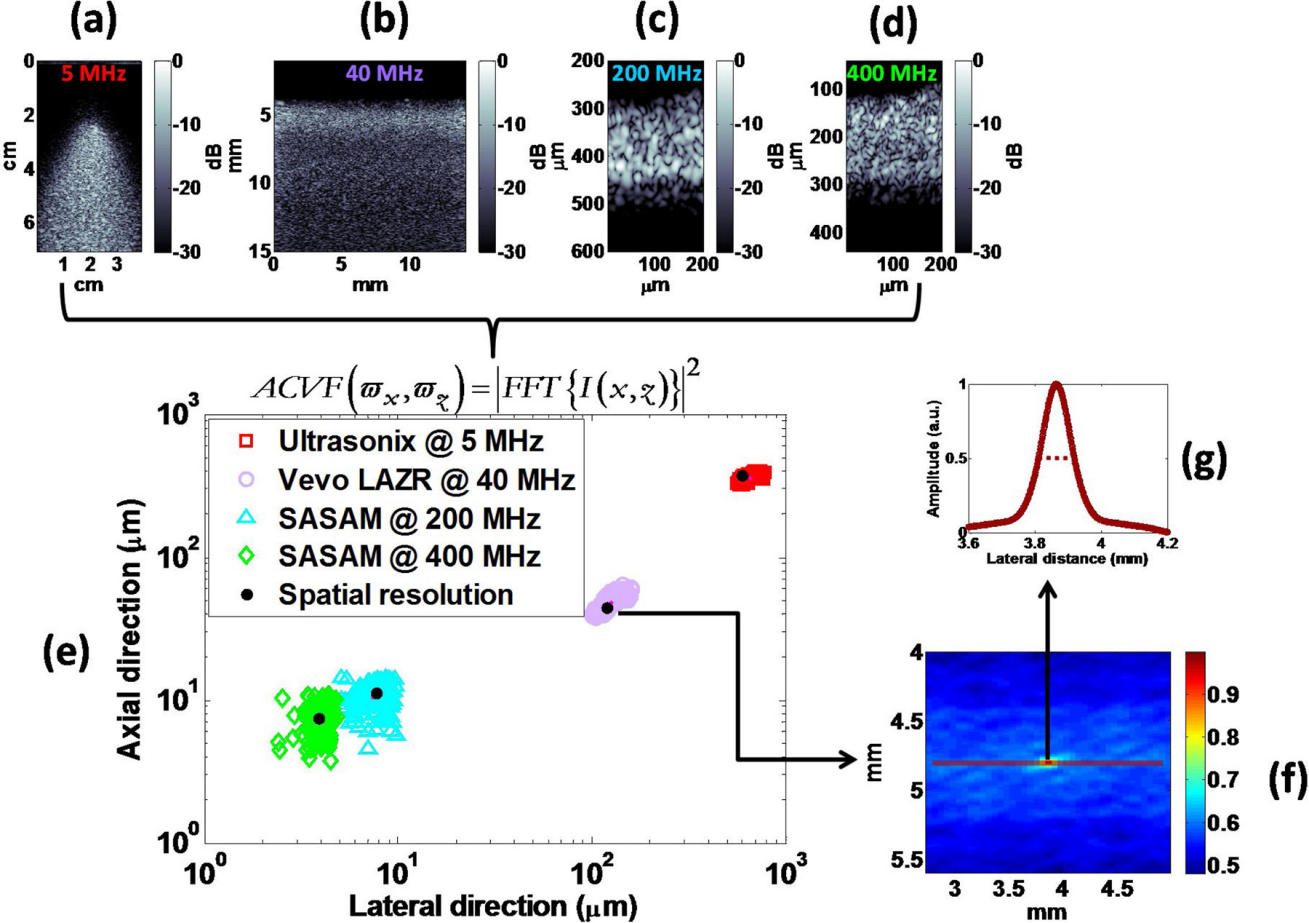
Effects of the imaging transducer on PA speckle size. PA B-mode images from speckle phantoms imaged with the (a) Ultrasonix RP, (b) VevoLAZR and the SASAM at (c) 200 and (d) 400 MHz. (e) Comparison between estimated speckle size and the spatial resolution of each system. (f) A representative 2D ACVF function from the VevoLAZR. (g) The lateral cross-section of the ACVF was used to estimate speckle size by measuring its FWHM.

Fig. 6 shows experimental results of PA speckle formation in imaging systems of multiple transducer frequencies (5–400 MHz). Speckle is ubiquitous to all images and extends from the centimeter (Ultrasonix RP, Fig. 6a) to the micrometer (SASAM, Fig. 6d) length scales. For the Ultrasonix RP (Fig. 6a), the large-scale pattern with speckle forms in accordance to the shape of the diffuse light distribution profile (see Supplementary Fig. 1a). Additionally, because of the limited-view geometry, speckle size changes as a function of depth. These effects are also present in the VevoLAZR system (Fig. 6b) with light emission from two rectangular strips oriented at 30° relative to the acoustic aperture [44]. SASAM images at 200 and 400 MHz (Fig. 6c and d, respectively) reveal different speckle patterns for the same phantom. The speckle size at 400 MHz is smaller than the 200 MHz image, consistent with the effects of transducer properties on image speckle.

Fig. 6e shows the speckle size estimations for all transducers. The speckle size is obtained from the 2D ACVF (Fig. 6f) along with the lateral profile of the ACVF maximum amplitude (Fig. 6g) shown for the 40 MHz probe. For each transducer, the speckle size was estimated from the FWHM of the horizontal and vertical line profiles through the center of the ACVF. The speckle size estimates for all systems were within 8.5% of the spatial resolution (see Supplementary Fig. 3). The speckle size in US imaging predominantly depends on the physical properties of the imaging transducer [41]. The transducer pulse bandwidth and beam width significantly affect the qualitative appearance of US speckle texture. As the size of the transducer focal zone decreased, the lateral width of the speckle, similar to US imaging. This was observed in images of the same phantom with two different transducers (Fig. 6c and d). Moreover, the calculated ACVF is comparable to the resolution volume [17], with axial speckle size inversely proportional to pulse bandwidth and the lateral speckle size increasing with range and beam width.

**Fig. 7:**
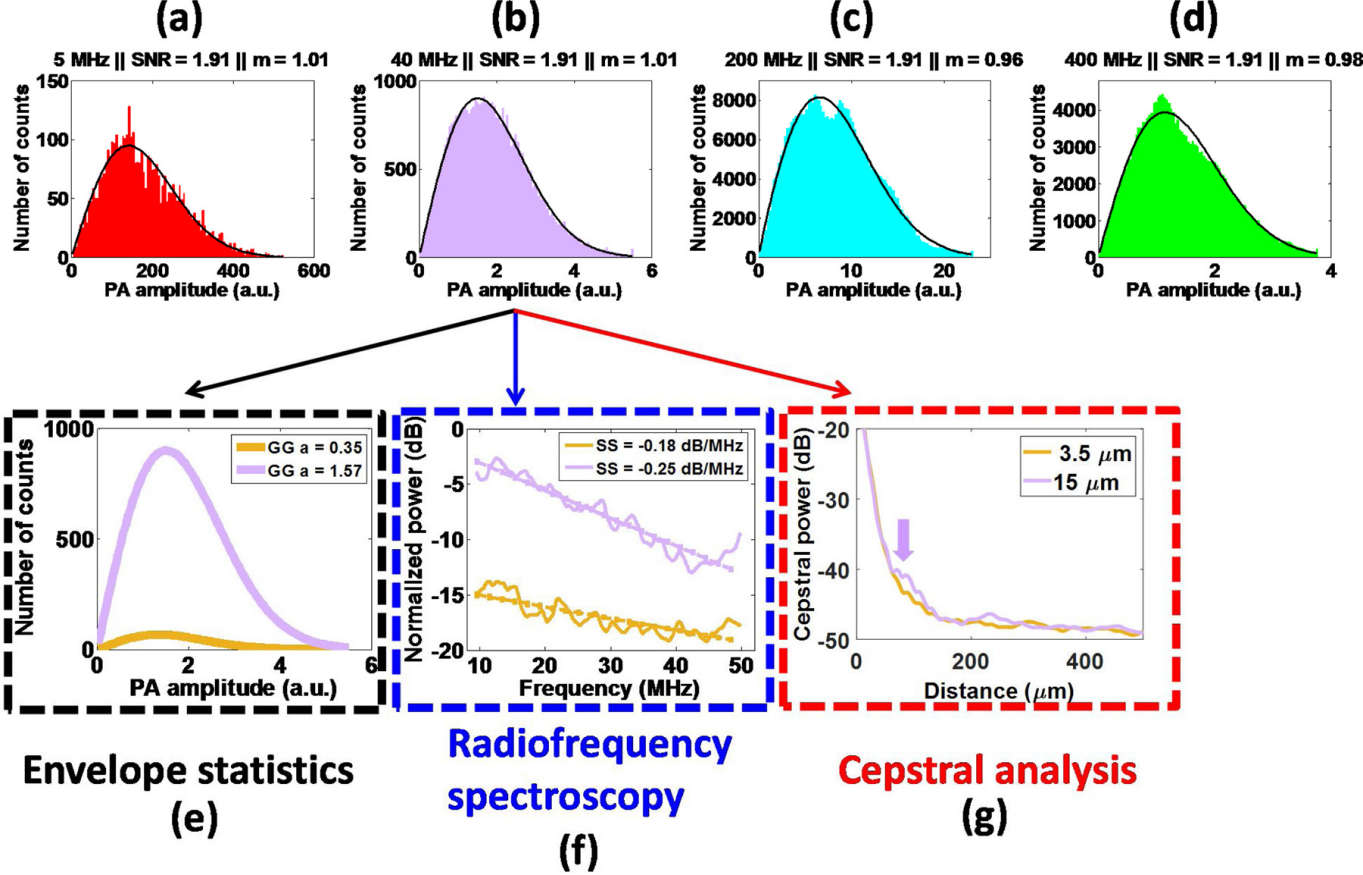
Speckle analysis techniques applied to experimental phantoms. Nagakami fits to the PA signal envelope for the phantoms imaged at (a) 5 MHz, (b) 40 MHz, (c) 200 MHz and (d) 400 MHz. The calculated SNR and Generalized Gamma m parameter are provided on top of each figure. For the VevoLAZR 40 MHz probe, the three analysis techniques presented in this paper, namely (e) Envelope statistics, (f) Radiofrequency spectroscopy and (g) Cepstral analysis were applied to phantoms consisting of 3 and 15 μm polystyrene beads. The arrow denotes a peak forming for the 15 μm absorber phantom.

Fig. 7 shows the application of the three analysis techniques (outlined in Fig. 2) to PA phantom images. The statistical distributions of the signal envelope histograms (Fig. 7a–d) establish that fully developed speckle formed across all the imaging systems spanning frequencies of 5–400 MHz. The criteria for fully developed speckle [40], namely the Rayleigh SNR equal to 1.91 and the Nakagami m parameter approaching 1, were met for all phantoms. This agrees with the simulation results (Fig. 3). When comparing two different sizes of absorbers (e.g. 3.5 and 15 μm), the Generalized Gamma a fit parameter to the envelope histograms (Fig. 7e) increases by 4.5x with increasing size. The PA SS of the normalized spectra decreases by a factor of 1.4x with increasing absorber size. The decrease in SS is consistent with the simulation results shown in Fig. 4. The power cepstra (Fig. 7f) reveal the presence of a peak for the 15 μm phantom around 220 μm while no discernable peak is visible for the phantom containing 3 μm beads. Even though these phantoms contain a randomized distribution of absorbers, it might be possible that periodicities might arise within the phantom for higher concentration of absorbers. The location of the cepstral peak would depend on spacing of the absorbers within one resolution volume [43]. Moreover, the prominence/amplitude of the peak would be affected by the degree of randomness in the distribution of absorbers. Comparing these peaks to the simulation results shown in Fig. 5c, one might postulate that the number density of periodically spaced absorber is most likely smaller than 15% in the entire phantom.

### 4.3 Photoacoustic speckle from tumor vasculature

#### 4.3.1 B-mode images from simulated and measured tumor blood vessels

**Fig. 8:**
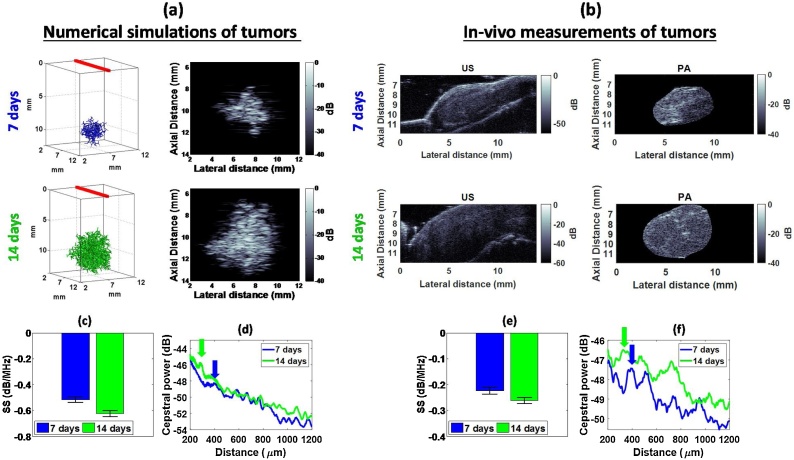
PA speckle from simulated and experimentally measured tumor vasculature. (a) Geometry of the vascular tree alongside the PA B-mode for the numerical simulations of breast tumor vasculature. The average vessel diameters used for the simulated vasculature were 37.8 μm and 48.2 μm at 7 and 14 days, respectively, and were all based on histological sectioning of in-vivo tumors. (b) US and PA images (750 nm) of in-vivo EMT-6 tumors at 7 (n = 3) and 14 (n = 4) days post-inoculation. (c)+(e) Spectral slope and (d)+(f) power cepstra for the numerical simulations of tumors modeling vascular growth and the in-vivo EMT-6 tumors. The arrows denote the location of the largest amplitude cepstral peaks.

Tumor angiogenesis is required to sustain the metabolic demands of the tumor growth [60]. The tumor vasculature typically does not have the hierarchical organization present in normal tissue [46]. Fractal models of the vasculature offer a means of studying vascular growth by focusing on the size, shape and orientation of each vessel while modeling the PA wave propagation from vascular trees. The effect of vessel size and number was studied using models of vascular architecture for breast tumors [8,9]. Fig. 8a shows the simulation geometry and PA B-mode images of simulated vascular trees at 7 and 14 days post-inoculation. Using histologically measured data of vessel diameters for breast tumors in mice [9], we simulated a representative subset of the vascular architecture. The simulated PA B-mode images of the vascular trees show the presence of speckle arising from several hundred non-resolvable vessels at various orientations contained within the imaging resolution volume. Much like the spherical absorbers shown in Fig. 3, Fig. 4, Fig. 5, Fig. 6, cylindrical PA sources also give rise to speckle when a sufficient number are contained within the resolution volume of the imaging transducer. Speckle is also present in the US and PA images of the in-vivo mouse tumor (Fig. 8b). It is present at all stages of mouse tumor growth vessel growth. Changes in the vasculature with tumor growth were modeled by increasing the branching order (and therefore the total number of vessels), consistent with previous in-vivo reports [49,50].

The US and PA speckle patterns are similar to each another for the in-vivo mouse tumors (Fig. 8b). Supplementary Fig. 4 shows that estimates of the speckle size for US and PA images of tumors are within 10% of the spatial resolution of the VevoLAZR transducer used to image these tumors. However, the speckle texture differs between PA simulations and experiments. While the experimental tumor speckle size is comparable to the transducer spatial resolution, the lateral size of the simulated speckle (estimated from Eq. (8)) is larger than the lateral resolution. This could be because the directivity of the linear array implemented in the simulations through Eq. (3) might differ from the true directivity of the transducer. Additionally, the effects of light fluence [61] and acoustic attenuation [62] on PA images are not taken into account, potentially affecting the pattern observed.

#### 4.3.2 Radiofrequency spectroscopy analysis from tumor vasculature

Fig. 8c–f show analysis of the growing tumor vasculature using radiofrequency spectroscopy and cepstral analysis. In simulations and in-vivo tumors, the PA SS (Fig. 8c and r) decreases by 20% and 17%, respectively as the tumor vasculature network grows in size from 7 to 14 days. The decrease in SS suggests that the average absorber size increases as the tumor grows [49]. Our previous work with vascular targeted treatments [9] has shown that the PA SS can be used to monitor changes in vessel size within tumors post vascular disrupting treatment. These results suggest that PA radiofrequency spectroscopy has the potential to differentiate changes in tumor vasculature (either due to tumor growth or treatment). This is an area of interest to multiple research areas, including drug discovery [63], where modalities such as PA imaging can have a translational impact.

#### 4.3.3 Cepstral analysis from tumor vasculature

Cepstral techniques can be used to probe the spatial arrangement of tumor blood vessels by identifying periodicities arising from non-resolvable absorbing structures. Fig. 8d and f show the average cepstra for simulated mouse xenograft breast tumor vasculature networks as well as in-vivo tumors. Both simulation and experiment contain an increasing number of total vessels from 7 to 14 days post-inoculation. As the overall number of vessels increased, the location of the most prominent (largest amplitude) peak in the cepstra decreased by 100 μm for both simulations (Fig. 8d) and experiments (Fig. 8f). The presence of ceptral peaks suggests the existence of periodicities in the vessel spatial distribution. These periodicities can arise due to specific vessel separation distances in the chaotic tumor vasculature being more common than others. Increasing the number of blood vessels in simulations or allowing the tumors to grow to 14 days (in-vivo) reduces the physical separation between each vessel. This is expected to result in a decrease in the mode (i.e. most commonly occurring) of the blood vessel separation distance. While tumor vasculature is known to lose much of its periodicity and order [46,48], it is possible that scant organization detectable with the cepstral analysis technique remains. While it may be the case that fewer than 10% of vessels have organized, periodic spacing, the results shown here point to differences between the periodic spacing of vessels in the 7 and 14-day old tumors.

According to the simulations and experiments, it might be possible to study the spatial separation of non-resolvable blood vessels in a tumor using cepstral analysis [43]. The PA signal detected is a superposition of PA waves, with greater contributions to the signal energy from vessels of the relevant length scale (as determined by the ultrasound wavelengths associated with the detection transducer bandwidth). The branching level of the vascular tree that would principally contribute to the PA signal is determined by a combination of the PA signal strength of the vessel size at that branching level and the detection bandwidth of the US transducer [64]. The former is itself a combination of the size and number of such vessels within the transducer resolution volume. Cepstral peaks could be associated with spacings at that branching level. Future studies will focus on understanding the location of cepstral peaks and how these are related to the structural organization of vessels within growing tumors [65].

### 4.4 Remarks on the nature of speckle in photoacoustic imaging

Our experimental findings show that PA speckle arises when PA waves from sub-resolution sources interfere. PA speckle is deterministic and can be described using speckle statistics. There are numerous references that describe PA imaging as a “speckle-free” modality [[24], [25], [26]]. The results of this work show that is not always the case. The presence of speckle in PA imaging depends on the imaging approach, as it is present in acoustic-resolution PA imaging but not in the optical resolution PA imaging. The pressure of the waves generated in PA imaging are more broadband compared to the backscattered waves in US imaging. The transducers and acquisition hardware act as lowpass or bandpass filters suppressing the high frequency components of the inherently broadband PA signals. In acoustic-resolution resolution PA imaging there are instances in which speckle is suppressed compared to the signal from boundaries. For example, when imaging individual blood vessels with diameters larger than the acoustic resolution volume, the vessel boundaries are accentuated, and the comparatively weak signal from speckle in the vessel interior cannot be readily appreciated. These effects are especially prominent in the cases of images with limited dynamic range [26] and occur in US imaging as well. However, as we demonstrate here, the inability to visualize PA speckle in such cases does not preclude its existence.

Formation of PA speckle from the tumor vasculature using limited-view geometries demonstrates the fundamental nature of the concept in-vivo. Much like the spherical absorbers shown in Fig. 1 through 6, cylindrical PA sources also give rise to speckle when a sufficient number are located within a resolution volume of the imaging transducer. PA speckle texture is also affected by acoustic attenuation [62] and light fluence [61] which were not incorporated into the theoretical model. Additionally, in-vivo tumors contain an unknown, but rather significantly large number of capillary beds [60] that would increase the number of non-resolvable PA absorbers in the transducer resolution volume. Moreover, for some imaging resolution volumes, vessels of the order larger than the resolution volumes exist. These parameters would influence the appearance of the speckle texture. Accounting for these effects would allow for more direct comparisons to experimentally measured tumors using limited-view PA geometries [[7], [8], [9],[66], [67], [68]]. The presence of speckle in PA imaging may be perceived as undesirable noise, as it is sometimes in US imaging, where it can obscure boundaries. However, since it is a deterministic signal, it has potential applications in the tissue characterization and speckle tracking [69].

## 5 Conclusions

Speckle in PA imaging arises from the spatiotemporal superposition of non-resolvable absorbers. In this work, we demonstrate that speckle carries information about the underlying absorber structure of individual sub-resolution sources. This paper introduced several analysis techniques (envelope statistics, radiofrequency spectroscopy and cepstral analysis) that can be applied to acoustic-resolution PA imaging with speckle. A fractal-based vascular model of tumors revealed that PA speckle also arises from complex tumor geometries consisting of cylindrical sources. These models were corroborated using PA data acquired from mouse tumors in-vivo. We also demonstrated the feasibility of the analysis techniques in quantifying absorber size and distribution. These findings have potential applications in monitoring changes in vessel size during vascular targeted cancer therapies.
