# Supplementary material for: Insights into photoacoustic speckle and applications in tumor characterization
Source: Photoacoustics. 2019 Apr 5;14:37–48. doi: 10.1016/j.pacs.2019.02.002 (PMC6505056; doi:10.1016/j.pacs.2019.02.002)
Supplement: Supplementary file 1 [file mmc1.docx]

**Supplementary information**

**Supplementary Fig. 1: PA imaging setups for the (a) Ultrasonix RP (5 MHz linear array, 128 elements) (b) VevoLAZR (40 MHz linear array, 256 elements). (c) Kibero SASAM (200 MHz and 400 MHz single element transducers).**

PA imaging systems

1. Ultrasonix RP clinical system (BK Ultrasound, Richmond, BC, Canada)

The laser source was an Nd:YAG-pumped optical parametric oscillator (OPO, Opotek Inc., Carlsband, CA, USA) operating at 760 nm. It was focused into the phantom using a focusing lens and a mirror tilted at 45^◦^. The laser was operated at a 10 Hz pulse repetition frequency (PRF), with each pulse emission triggering the acquisition of a passive 5 MHz center frequency linear array transducer with 7 MHz -6 dB bandwidth. The non-beamformed PA signals were acquired using an external DAQ attached to the RP unit. A total of 15 frames were acquired and beamformed offline using a delay-and-sum approach prior to temporal averaging of all frames at 14 Hz frame rate.

1. VevoLAZR pre-clinical system (Fujifilm-VisualSonics Inc., Toronto, ON, Canada)

A linear array probe with 256 elements and 40 MHz center frequency (41 MHz -6 dB bandwidth) with an integrated laser operating at 680 nm was used to image the phantom through water. The laser source was a Nd:YAG pumped OPO (Opotek Inc.) operating at 10 Hz PRF [33]. The light is emitted from two rectangular strips on both sides of the acoustic aperture of the transducer, at a 30^◦^ angle relative to the imaging plane. A total of 5 non-beamformed PA frames were acquired, beamformed and averaged offline.

1. Kibero SASAM photoacoustic microscope (Kibero GmbH, Saarbrucken, Germany)

The SAarland Scanning Acoustic Microscope (SASAM) was used with two different single element focused transducers operating at center frequencies of 200 MHz (aperture diameter/focal depth 500 μm, 138 MHz -6 dB bandwidth) and 400 MHz (aperture diameter/focal depth 350 μm, 218 MHz -6 dB bandwidth). A fiber-coupled 532 nm Nd:YAG laser (Teem Photonics, Meylan, France) operating at 4 kHz PRF was used to generate the PA waves within the phantom. A variable neutral density (ND) filter (Thorlabs Inc., Newton, NJ, USA) was used to vary the energy at the input end of a single mode fiber (Costal Connections, Venura, CA, USA). The laser output was passed through a beam splitter; the transmitted portion was focused onto the phantom using a 4X microscope objective, and the reflected portion was directed into a Mach6 energy meter (Gentec Electro-Optics Inc., Quebec City, QC, Canada). The transducer and laser were coaxially aligned and a phantom region of$200\mu m\times100\mu m$ was scanned through the confocal zone. A total of five B-mode images were acquired $20\mu m$ apart and were spatially averaged. The same phantom was imaged using both the 200 and 400 MHz transducers.

PA imaging systems characteristics

**Supplementary Fig. 2: (a) Summary of the characteristics of the phantoms and the PA imaging system. (b) Spatial resolution of each transducer as a function of its center frequency. (c) PA frequency response for the transducers using in this study measured using a 200 nm gold film.**

Phantom preparation

Phantoms imaged by the linear arrays were prepared using 10% (w/v) porcine skin gelatin while the phantom imaged by the single element system consisted of 1.5% (w/v) agarose (Sigma-Aldrich Co., St. Louis, MO, USA) as the suspending medium. The concentration of gelatin was chosen in order to achieve a frequency dependent attenuation of approximately$0.25db/cm\cdot MHz$. The optical absorbers for each system were: (i) 5 MHz linear array:$250\mu m$black glass beads (Corpuscular Inc., Cold Spring, NY, USA); (ii) 40 MHz linear array:$15\mu m$black polystyrene beads (Merck Millipore, Pithiviers, France); (iii) 200/400 MHz single elements:$0.2\mu m$ black polystyrene beads (Polysciences Inc., Warrington, PA, USA). The number of beads per each phantom was calculated based on the criterion that at least 10 beads per resolution volume of each imaging transducer.

In order to construct the phantoms imaged by the linear array systems, degassed water was heated to 35^◦^C along with the beads before the gelatin powder was slowly added. This suspension was magnetically stirred in order to achieve homogeneity until it reached 65^◦^C and the gelatin was entirely dissolved. It was then placed inside a custom-made rotisserie for 3 hours at room temperature to ensure homogenous mixing of the beads inside each phantom. Each phantom was then kept at 4^◦^C for 24 hours prior to imaging in order to achieve full solidification of the gelatin. The $0.2\mu m$ beads for the single element transducer phantom were centrifuged, supernatant aspirated and then re-suspended in molten agarose. The agarose was then sandwiched between two microscope coverslips and kept at 4^◦^C for 15 minutes.

**Supplementary Fig. 3: Speckle size estimations and comparison with spatial resolution for (a) the Ultrasonix RP, (b) the VevoLAZR and the Kibero SASAM at (c) 200 MHz and (d) 400 MHz in the axial and lateral directions. Each symbol represents the average speckle size estimated from a sliding window ROI through each image. Cross bars represent the standard deviation of the mean speckle size estimates in both dimensions.**

**Supplementary Fig. 4: Ultrasound and photoacoustic speckle size estimates for a representative Balb/c mouse EMT-6 tumor imaged 14 days post-inoculation. The images were acquired with the VevoLAZR system at 40 MHz and 750 nm illumination. Each circle represents the average speckle size estimated from a sliding window ROI through each image. Cross bars represent the standard deviation of the mean speckle size estimates in both dimensions.**
